# Androgen excess disorders remain undiagnosed in one of every four premenopausal women with Type 1 diabetes

**DOI:** 10.1093/hropen/hoaf048

**Published:** 2025-07-15

**Authors:** Ane Bayona Cebada, Lía Nattero-Chávez, Esther De la Calle De la Villa, Alejandra Quintero Tobar, Sara de Lope Quiñones, Beatriz Dorado Avendaño, Tom Fiers, Jean-Marc Kaufman, Manuel Luque-Ramírez, Héctor F Escobar-Morreale

**Affiliations:** Department of Endocrinology and Nutrition, Hospital Universitario Ramón y Cajal, Madrid, Spain; Diabetes, Obesity & Human Reproduction Research Group, Instituto Ramón y Cajal de Investigación Sanitaria (IRYCIS) & Centro de Investigación Biomédica en Red de Diabetes y Enfermedades Metabólicas Asociadas (CIBERDEM), Madrid, Spain; Department of Medicine and Medical Specialties, Universidad de Alcalá, Alcalá de Henares, Spain; Department of Endocrinology and Nutrition, Hospital Universitario Ramón y Cajal, Madrid, Spain; Diabetes, Obesity & Human Reproduction Research Group, Instituto Ramón y Cajal de Investigación Sanitaria (IRYCIS) & Centro de Investigación Biomédica en Red de Diabetes y Enfermedades Metabólicas Asociadas (CIBERDEM), Madrid, Spain; Department of Endocrinology and Nutrition, Hospital Universitario Ramón y Cajal, Madrid, Spain; Department of Medicine and Medical Specialties, Universidad de Alcalá, Alcalá de Henares, Spain; Diabetes, Obesity & Human Reproduction Research Group, Instituto Ramón y Cajal de Investigación Sanitaria (IRYCIS) & Centro de Investigación Biomédica en Red de Diabetes y Enfermedades Metabólicas Asociadas (CIBERDEM), Madrid, Spain; Diabetes, Obesity & Human Reproduction Research Group, Instituto Ramón y Cajal de Investigación Sanitaria (IRYCIS) & Centro de Investigación Biomédica en Red de Diabetes y Enfermedades Metabólicas Asociadas (CIBERDEM), Madrid, Spain; Department of Endocrinology and Nutrition, Hospital Universitario Ramón y Cajal, Madrid, Spain; Laboratory for Hormonology, Ghent University Hospital, Ghent, Belgium; Department of Endocrinology, Ghent University Hospital, Ghent, Belgium; Department of Endocrinology and Nutrition, Hospital Universitario Ramón y Cajal, Madrid, Spain; Diabetes, Obesity & Human Reproduction Research Group, Instituto Ramón y Cajal de Investigación Sanitaria (IRYCIS) & Centro de Investigación Biomédica en Red de Diabetes y Enfermedades Metabólicas Asociadas (CIBERDEM), Madrid, Spain; Department of Medicine and Medical Specialties, Universidad de Alcalá, Alcalá de Henares, Spain; Department of Endocrinology and Nutrition, Hospital Universitario Ramón y Cajal, Madrid, Spain; Diabetes, Obesity & Human Reproduction Research Group, Instituto Ramón y Cajal de Investigación Sanitaria (IRYCIS) & Centro de Investigación Biomédica en Red de Diabetes y Enfermedades Metabólicas Asociadas (CIBERDEM), Madrid, Spain; Department of Medicine and Medical Specialties, Universidad de Alcalá, Alcalá de Henares, Spain

**Keywords:** hirsutism, hyperandrogenism, ovulatory dysfunction, polycystic ovary syndrome, Type 1 diabetes

## Abstract

**STUDY QUESTION:**

How frequent are androgen excess disorders, including polycystic ovary syndrome (PCOS), among women with Type 1 diabetes mellitus (T1D)?

**SUMMARY ANSWER:**

One in every four women with T1D suffer from undiagnosed androgen disorders, with the classic phenotype of PCOS being the most frequent.

**WHAT IS KNOWN ALREADY:**

Systemic iatrogenic hyperinsulinism is unavoidable in patients with T1D because insulin is administered subcutaneously instead of being secreted directly into the portal circulation. Since insulin acts as a co-gonadotrophin at the ovary, iatrogenic hyperinsulinism might trigger androgen secretion in predisposed women. Most studies conducted to date have reported increased prevalences of androgen excess disorders in premenopausal women with T1D, yet these studies were hampered by methodological limitations that preclude reaching a definite conclusion on the issue.

**STUDY DESIGN, SIZE, AND DURATION:**

From January 2020 to March 2024, we conducted a cross-sectional study including women with T1D.

**PARTICIPANTS, SETTING, METHODS:**

We recruited 149 consecutive premenopausal women with T1D who attended the diabetes clinics of an Academic Hospital at Madrid, Spain. We compared them with 295 typical patients with PCOS who did not have T1D. We used state-of-the-art mass spectrometry techniques to measure serum androgens and equilibrium dialysis to measure free testosterone and followed the latest guidelines to phenotype patients.

**MAIN RESULTS AND THE ROLE OF CHANCE:**

Hyperandrogenic disorders (considering PCOS, idiopathic hyperandrogenism, and idiopathic hirsutism as a whole) were present in 39 (of 149) women with T1D (26%, 95% CI: 20–34%), including 30 women who fulfilled the PCOS diagnostic criteria, indicating a prevalence of 20% (95% CI: 15–27%). The most common PCOS phenotype was the classic combination of hyperandrogenism and ovulatory dysfunction. Women with T1D and PCOS were younger (mean age 25 ± 7 vs 31 ± 9 years-old, *P *= 0.003) and their onset of T1D was more frequently premenarcheal (73% vs 46%, *P *= 0.008) compared to those without PCOS. Compared to 295 typical patients with PCOS without T1D, the 30 women with T1D and PCOS showed milder hyperandrogenic signs and lower free testosterone concentrations [13 (9, 25) vs 21 (15, 29) pM, *P *< 0.001] regardless of the glucose tolerance of the former.

**LIMITATIONS, REASONS FOR CAUTION:**

We acknowledge the possibility of selection bias: having excluded T1D women already diagnosed with PCOS, we may have underestimated actual prevalence rates. Also, the cross-sectional design of the study precluded us from obtaining any causality insights about the associations found here.

**WIDER IMPLICATIONS OF THE FINDINGS:**

One in every four women with T1D suffer androgen excess disorders, with the classic combination of hyperandrogenism and ovulatory dysfunction being the most common phenotype of PCOS. Women with a premenarcheal onset of T1D are particularly susceptible to developing androgen excess disorders and may benefit from future preventive measures at young ages. Routine screening for these prevalent disorders seems reasonable to avoid the negative consequences of androgen excess and chronic ovulatory dysfunction on the general and reproductive health of T1D women.

**STUDY FUNDING/COMPETING INTEREST(S):**

This work was supported by grants PIE18/01122 and PI21/00116 from Instituto de Salud Carlos III, and co-funded by the European Union. A.B.C. is the recipient of a Río Hortega grant (CM19/00138) from Instituto de Salud Carlos III. CIBERDEM and IRYCIS also belong to Instituto de Salud Carlos III. The funding source was not involved in the study design, the data collection, analysis and interpretation, nor in the decision to submit the paper for publication. The authors have no competing interests to disclose.

**TRIAL REGISTRATION NUMBER:**

N/A.

WHAT DOES THIS MEAN FOR PATIENTS? Reproductive complaints are common in women with Type 1 diabetes (T1D), even in those with good metabolic control through insulin administration. Disorders caused by an excess of androgen in women, such as polycystic ovary syndrome (PCOS), might underlie these symptoms.

The aim of this work was to determine the frequency of androgen excess disorders, including PCOS, in premenopausal women with T1D during their reproductive years. We also aimed to establish the characteristics of PCOS in this population and to analyze which particular risk factors might trigger the development of androgen excess and PCOS in these women.

Our study found that one in every four women with T1D suffered from an androgen excess disorder without knowing it, and those diagnosed with T1D at a younger age were particularly susceptible to developing an androgen excess disorder. However, compared to 295 non-T1D patients with PCOS, the women with T1D and PCOS showed milder signs and lower androgen levels.

Routine screening for these prevalent disorders seems reasonable to avoid the negative consequences of androgen excess and long-term ovulatory dysfunction on the general and reproductive health of women with T1D.

## Introduction

For decades, reproductive abnormalities have characterized women with Type 1 diabetes (T1D) and very poor metabolic control (Bergqvist, 1954). After the widespread application of intensive insulin therapy, features of functional hyperandrogenism and/or menstrual irregularities have been increasingly recognized in women with T1D even with adequate metabolic control (Codner *et al.*, 2012; Thong *et al.*, 2020). Prevalence rates of polycystic ovary syndrome (PCOS) in women with T1D vary between 2 and 49% (Escobar-Morreale and Roldán-Martín, 2016; Bayona *et al.*, 2022; García-Sáenz *et al.*, 2023), while related traits such as oligomenorrhea, hyperandrogenemia, and polycystic ovarian morphology (PCOM) are also common (Escobar-Morreale and Roldán-Martín, 2016; Bayona *et al.*, 2022). Whether these associations confer an additional cardiovascular risk or have an impact on metabolic control needs clarification.

PCOS is a common endocrine disorder in premenopausal women, with prevalence rates between 5 and 15% in the general population (Teede *et al.*, 2023). The association between T1D and PCOS was first reported in the Spanish population in the year 2000 (Escobar-Morreale *et al.*, 2000). Our 2022 meta-analysis showed a pooled PCOS prevalence of 26% (95% CI: 19–35%; 13 studies) among 684 women with T1D, yet heterogeneity between the studies was high (Bayona *et al.*, 2022). The inconsistency regarding reported rates of PCOS may lie not only in the methodology used in individual studies but also in the intrinsic complexity and heterogeneity of this perplexing disorder. In addition, the definition and diagnosis of PCOS have changed recently; current recommendations advocate for specifying the phenotype when diagnosing PCOS (Teede *et al.*, 2023). To the best of our knowledge, no publications have reported the prevalence of PCOS or of its different phenotypes in women with T1D.

The prevalence of PCOS in women with T1D might have been overestimated in previous studies for reasons related to the measurement of serum androgen concentrations. Total and free testosterone (FT) concentrations are largely dependent on sex hormone-binding globulin (SHBG) concentrations, and the hepatic synthesis and secretion of this globulin are markedly different in women with T1D compared to those in the general population. Under physiological conditions, SHBG concentrations are inhibited by insulin and adipokines secreted into the portal circulation, explaining its reduced concentrations in people with obesity (Simó *et al.*, 2015) and in women with PCOS from the general population (Escobar-Morreale and San Millán, 2007). Such a decrease in SHBG levels further contributes to the increase in FT concentrations that characterize the latter (Roldán *et al.*, 2001).

On the contrary, in women with T1D and PCOS, hyperinsulinism is necessarily related to exogenous insulin, since pancreatic insulin secretion is negligible. Delivery of insulin by a non-physiological subcutaneous route determines that exogenous insulin circulates through the systemic circulation before reaching the portal circulation at the levels needed to suppress hepatic glucose output. On the contrary, in healthy people, endogenous insulin levels in the systemic circulation are much lower because insulin directly reaches the liver after being secreted by the pancreas, and, as a consequence, insulin gets into the systemic circulation only after substantial hepatic clearance. Therefore, in women with T1D, the ovaries are necessarily exposed to excess insulin, increasing androgen secretion and enlarging ovarian volume in predisposed women (Roldán *et al.*, 2001; Escobar-Morreale *et al.*, 2021). However, because the insulin quantities that reach the liver are those needed to suppress glucose output and are necessarily close to the physiological range to avoid hyper- and hypoglycemia, iatrogenic hyperinsulinism does not inhibit hepatic SHBG secretion (Roldán *et al.*, 2001; Escobar-Morreale *et al.*, 2021). Therefore, the normal SHBG levels observed in women with T1D may ameliorate the increase in FT even in the presence of androgen excess and might contribute to their relatively mild hyperandrogenic symptoms (Roldán *et al.*, 2001).

To date, studies conducted in T1D used reference ranges for circulating androgens derived from the general population, yet such reference ranges might be inadequate for women with T1D. Calculation of FT from testosterone and SHBG concentrations is currently recommended for the diagnosis of PCOS, because it correlates well with direct FT measured by equilibrium dialysis (Vermeulen *et al.*, 1999); this may not be applicable to women with T1D. Furthermore, current evidence-based practice guidelines for the management of PCOS recommends the use of validated accurate liquid chromatography–tandem mass spectrometry assays (LC–MS/MS) over direct immunoassays for diagnosing biochemical hyperandrogenism, particularly because the use of immunoassays overestimate androgen concentrations leading to misdiagnosis (Teede *et al.*, 2023; Luque-Ramírez *et al.*, 2025). Only one earlier study addressing the prevalence of PCOS in women with T1D used LC–MS/MS techniques for androgen measurement (Gunness *et al.*, 2018).

Hence, the aim of this study was to determine the prevalence of disorders of functional androgen excess (including idiopathic hyperandrogenism, idiopathic hirsutism, and PCOS) and of other related hyperandrogenic traits in a cohort of premenopausal women with T1D, while addressing current caveats in knowledge. Also, we aimed to establish the phenotypic characteristics of PCOS in this population, and, because not all women with T1D develop PCOS, we analyzed which factors, such as total daily insulin dose, premenarcheal diagnosis of T1D or presence of overweight, might trigger the development of androgen excess and PCOS in these women (Bayona *et al.*, 2022; García-Sáenz *et al.*, 2023).

## Materials and methods

### Ethical approval

The study protocol was approved by the ethics committee of Hospital Universitario Ramón y Cajal (approval on 9 March 2020; protocol ID: 045/20). Informed consent was obtained from all participants before recruitment.

### Study design and setting

From January 2020 to March 2024, we recruited consecutively all women aged 18–45 years diagnosed with diabetes mellitus Type 1a (American Diabetes Association Professional Practice Committee *et al.*, 2024) for at least 1 year, when reporting to our diabetes clinics. All patients were on intensive insulin therapy, and inclusion criteria required that women with T1D had a gynecological age of at least 2 years (Ibáñez *et al.*, 2017), and that none of them had been previously diagnosed with PCOS or other related disorders. Exclusion criteria were: (i) the ‘honeymoon’ period of T1D; (ii) overt thyroid dysfunction, hyperprolactinemia, or congenital adrenal hyperplasia (CAH); (iii) use of oral contraceptives during the previous 3 months; (iv) ongoing pregnancy; or (v) severe chronic disease, including genetic syndromes and end-stage renal disease (KDIGO 4 or 5 or kidney transplant).

### Phenotyping of subjects included in the study

We reviewed the medical records of the patients and collected clinical parameters related to T1D. Data from 14- or 10-day ambulatory glucose profiles (AGPs) were recorded following the recommendations of the International Consensus on Time in Range (Battelino *et al.*, 2019), as well as current daily insulin dose (units per kilogram and day, U/kg/d). Waist and hip circumferences (cm), height (m), and weight (kg) served to calculate waist-to-hip ratio (WHR) and BMI (kg/m^2^). The average of the three measurements was used as an estimation of office systolic and diastolic blood pressure (BP) readings. Hirsutism was defined as the presence of excessive terminal hair in typically masculine areas by a modified Ferriman–Gallwey (mFG) score ≥ 8 (Hatch *et al.*, 1981). Other signs such as acne vulgaris or female pattern hair loss were considered as signs of hyperandrogenism only when accompanied by increased serum androgens.

We compared women with T1D and PCOS with their non-PCOS counterparts and with age-matched typical women with PCOS belonging to a historical cohort of PCOS patients without T1D (Luque-Ramírez *et al.*, 2025), who presented with either abnormal glucose tolerance (n = 45) or normoglycemia (n = 250).

All women included in the study were sampled during the follicular phase (between days 2 and 8) after a spontaneous or progesterone withdrawal-induced menstruation. Blood samples were withdrawn between 8 and 10 am after a 10- to 12-h fasting period for measuring fasting plasma glucose, renal and hepatic function, serum lipid profile, glycated hemoglobin (HbA_1c_), complete blood count and coagulation tests, ultrasensitive C-reactive protein (CRP), iron tests, and hormonal analysis including thyrotropin (TSH), FSH, LH, prolactin, 17β-estradiol, SHBG, and dehydroepiandrosterone sulfate (DHEA-S), as well as basal and 30 min cosyntropin-stimulated 17-hydroxyprogesterone (17-OHP).

Aliquots of serum samples were stored at −80°C until assayed for total testosterone (TT) and Δ^4^-androstenedione (Δ^4^) by LC–MS/MS at the Laboratory for Hormonology of the University of Ghent, Belgium, using an AB Sciex 6500 triple-quadrupole mass spectrometer (AB Sciex, Toronto, Canada). The lower limit of quantification (LLOQ) was 0.04 nM (1.2 ng/dl) for TT, and the inter-assay coefficient of variability (CV) was 8.3% at 1.27 nM (36.7 ng/dl) and 3.1% at 10.7 nM (307.8 ng/dl). Serum LLOQ was <0.17 nM (0.05 ng/ml) for Δ^4^, and the interassay CV was <7.1%. FT was measured using an equilibrium dialysis method (Fiers *et al.*, 2018). In brief, 1 ml of undiluted serum was dialyzed against a protein-free buffer using FastMicro-Equilibrium dialyzer cartridges equipped with regenerated cellulose membranes (25 kDa, Harvard Apparatus, Holliston, MA, USA). The dialysis process was conducted at 37°C for 24 h at a pH of 7.28. Following equilibrium dialysis, FT concentrations in the dialysate were measured by LC–MS/MS. The interassay CV for FT measurement was 13.5% at 6.2 pmol/l (0.18 ng/dl), with a limit of quantitation of 2.4 pmol/l (0.07 ng/dl).

Other hormonal analyses were performed by our local laboratory. DHEA-S was measured using Advia Centaur (Siemens Healthcare Sector, Erlangen, Germany), with an LLOQ of 0.081 μM (3 ug/dl). 17-OHP was measured by ELISA (DRG Instruments GmbH, Marburg, Germany), with an LLOQ of 0.103 nM (0.034 ng/ml) and a range of the assay between 0.103 and 60 nM (0.034–20 ng/ml). SHBG was measured using an automated immunoassay (IMMULITE 2000, Siemens Healthcare Sector, Erlangen, Germany) with an LLOQ of 0.02 nM and mean intra-assay and inter-assay CVs <10%. TSH, FSH, LH, prolactin, and 17β-estradiol were measured using Alinity i immunoassays (Abbott Diagnostics, Chicago, IL, USA). Prolactin was assayed in two consecutive samples (obtained immediately and after 15 min of placing an intravenous line) to exclude a possible interference of venipuncture stress. Those levels of prolactin that remained elevated in the 15-min determination were pretreated with polyethylene glycol to exclude the presence of macroprolactinemia.

Either ovarian ultrasound or serum anti-Müllerian hormone (AMH) levels were used to define PCOM if needed for diagnosing PCOS when either the hyperandrogenism or the oligoovulation criteria were not met by the patient (Teede *et al.*, 2023). In women in whom ovarian ultrasound was not available, serum AMH levels were assayed. AMH was measured using an automated immunoassay on a Cobas e601^®^ analyzer (Elecsys^®^, Roche Diagnostics, Mannheim, Germany). The assay limits of detection and LLOQ were 0.07 and 0.21 pM (0.01 and 0.03 ng/ml), respectively. The intra-assay and inter-assay CVs were <4%. The limit above the measuring range was 164 pM (23 ng/ml). For AMH assays, the upper limit of normality was defined by a value above the 95th percentile (>55.9 pM or 7.8 ng/ml) of a local control group composed of 91 non-hyperandrogenic premenopausal female volunteers presenting with regular menses; their age (29 ± 6 years) and BMI (26.6 ± 7.6 kg/m^2^) were similar to that of our patients (Luque-Ramírez *et al.*, 2025). An elevated AMH may reclassify patients with ovulatory dysfunction and isolated hyperandrogenism into a non-hyperandrogenic or an ovulatory phenotype of PCOS, respectively (Teede *et al.*, 2023).

Finally, to assess insulin sensitivity in patients with T1D, a score was estimated following the model proposed by Williams *et al.* (2000), which includes WHR, presence of hypertension, and HbA_1C_ levels; a cut-off value of estimated glucose disposal rate (eGDR) of <6.4 mg/kg × min^−1^ was used to diagnose insulin resistance.

We established not only the diagnosis of PCOS (i.e. women meeting two of these three criteria: clinical and/or biochemical hyperandrogenism, ovulatory dysfunction, and PCOM) but also its specific phenotype (Azziz *et al.*, 2019; Teede *et al.*, 2023). Phenotype 1 (classic PCOS) was defined as the presence of hyperandrogenism, ovulatory dysfunction, and PCOM; Phenotype 2 (also deemed classic PCOS) included the presence of hyperandrogenism and ovulatory dysfunction in the absence of PCOM. Phenotype 3 or ovulatory PCOS consisted of hyperandrogenism and PCOM. Phenotype 4 or non-hyperandrogenic PCOS was defined as ovulatory dysfunction and PCOM.

Hyperandrogenemia refers to an increase in serum androgen levels above the upper reference range, mainly TT or FT, with or without a mild elevation of DHEA-S or Δ^4^. For LC–MS/MS measurements (TT, FT, and Δ^4^), biochemical hyperandrogenism and upper reference range were defined by the presence of values above 95th percentile of a sample of non-hyperandrogenic premenopausal female volunteers presenting with regular menses (Luque-Ramírez *et al.*, 2025). For equilibrium dialysis FT, the 95th percentile was calculated in the subgroup of T1D women without ovulatory dysfunction or dermo-cosmetic issues (n = 91). For DHEA-S, we used a local reference range as described elsewhere (Alpañés *et al.*, 2015).

Ovulatory dysfunction was defined as clinical evidence of menstrual cycle irregularity, including length of menstrual cycles <21 days or >35 days, <8 cycles per year (oligomenorrhea), or the absence of menstruation for at least 3 months within the last year (amenorrhea) (Munro *et al.*, 2022; Teede *et al.*, 2023). PCOM was defined qualitatively (presence or absence) or quantitatively by a follicle number per ovary ≥20, an ovarian volume ≥10 ml, or a follicle number per section ≥10 in at least one ovary (Dewailly *et al.*, 2014; Teede *et al.*, 2023).

### Sample size calculation and statistical analysis

Considering a 40.5% prevalence of PCOS in young women with T1D when including all PCOS phenotypes as a whole (Codner *et al.*, 2006), and a 95% confidence interval, an accuracy of 5%, and a global population of ∼3000 patients with T1D followed at our diabetes outpatients clinic (of whom ∼33.1% correspond to premenopausal women) (Nattero-Chávez *et al.*, 2023), we estimated a sample size of 150 patients, considering a replacement rate of 10% (http://epitools.ausvet.com.au).

Data are shown as the mean ± SD and 95% CI (lower limit–upper limit) or median (25th percentile, 75th percentile) for continuous variables, and as counts (%) for categorical variables. To quantify the agreement between equilibrium dialysis and calculated FT beyond simple correlation analysis, we calculated Lin’s Concordance Correlation Coefficient (CCC), and a Bland–Altman plot was generated.

For continuous variables, normal distribution was assessed using the Shapiro–Wilk test. We applied nonparametric tests to variables that did not follow the normal distribution. The comparison between T1D-PCOS and T1D without PCOS groups was performed using Student’s *t*-test or Mann–Whitney *U*-test for continuous variables as appropriate, and by *X*^2^ or Fisher’s tests for qualitative variables. Metabolic parameters and complications-related variables were adjusted by age of study and duration of T1D using regression models. To compare continuous variables across three groups (T1D-PCOS, abnormal glucose tolerance-PCOS, and normoglycemia-PCOS), we used general linear models while adjusting for BMI. The margins command was used to compute predicted means for each group, and pairwise comparisons between groups were performed. Statistical significance was defined as a *P*-value <0.05 for all tests.

## Results

### Study population characteristics and definition of hyperandrogenism in T1D

Of the 151 patients recruited, two were excluded due to diagnoses of non-classic CAH and hyperthyroidism, respectively; therefore, 149 women with T1D were included in the final analysis. Their characteristics are shown in Table 1. The onset of T1D was premenarcheal in approximately half of them, all received intensive insulin therapy, and their mean HbA_1C_ was 7.5 ± 1.5%.

**Table 1.** Clinical, anthropometric, and laboratory parameters for T1D patients considered as a whole, women with T1D and PCOS and women with T1D but without PCOS.

Variables	All T1D patients (n = 149)	T1D and PCOS (n = 30)	T1D without PCOS (n = 119)	*P* value	**Adjusted *P* value** *
Age (years)	30 ± 9	25 ± 7	31 ± 9	**0.003**	–
Duration of T1D (years)	14.5 ± 8.9	14.3 ± 5.9	14.6 ± 9.6	0.641	–
Age at diagnosis of T1D (years)	15 ± 10	11 ± 7	16 ± 10	**0.010**	–
Insulin therapy: CSII	37 (24.8%)	11 (36.7%)	26 (21.8%)	0.093	–
Total daily insulin dose (UI/kg/day)	0.63 ± 0.24	0.70 ± 0.34	0.61 ± 0.20	0.338	0.380
Age of menarche (years)	13.0 ± 1.7	13.3 ± 1.9	12.9 ± 1.7	0.216	–
Premenarcheal diagnosis of T1D	77 (51.7%)	22 (73.3%)	55 (46.2%)	**0.008**	–
Family history of PCOS	21 (14.1%)	3 (10.0%)	18 (15.1%)	0.471	–
Family history of Type 2 diabetes	91 (61.1%)	21 (70.0%)	70 (58.8%)	0.262	–
Hypertension	11 (7.3%)	1 (3.3%)	10 (8.4%)	0.695	0.782
Microangiopathy	16 (10.7%)	4 (13.3%)	12 (10.1%)	0.607	0.179
Macroangiopathy	1 (0.6%)	0	1 (0.8%)	0.614	–
Estimated GDR (mg/kg · min^−1^)	10.3 ± 1.6	10.1 ± 1.5	10.3 ± 1.6	0.463	0.374
Systolic blood pressure (mmHg)	113 ± 13	110 ± 13	114 ± 13	0.112	0.259
Diastolic blood pressure (mmHg)	72 ± 9	68 ± 7	73 ± 9	**0.008**	**0.030**
BMI (kg/m^2^)	24.2 ± 4.3	23.6 ± 4.1	24.4 ± 4.4	0.500	–
Waist-to-hip ratio	0.78 ± 0.05	0.80 ± 0.06	0.78 ± 0.05	0.075	–
Hirsutism score	2 (1, 4)	3 (2, 6)	2 (1, 4)	**0.001**	–
Hirsutism	6 (4.0%)	4 (13.3%)	2 (1.7%)	**0.015**	–
Ovulatory dysfunction	56 (38.5%)	27 (90%)	29 (24.3%)	**<0.001**	–
Polycystic ovarian morphology	31/129 (24.0%)	23/28 (82.1%)	8/101 (7.9%)	**<0.001**	–
Time in range 3.9–10.0 mM (%)	61.1 ± 19.7	60.4 ± 16.9	61.3 ± 20.5	0.793	0.779
Time in hypoglycaemia <3.9 mM (%)	5.4 ± 4.6	7.0 ± 5.6	4.8 ± 4.2	**0**.**019**	0.090
Time in hyperglycaemia >10.0 mM (%)	33.4 ± 19.7	32.6 ± 17.5	33.6 ± 20.2	0.910	0.517
Coefficient of variation	37.9 ± 8.0	41.0 ± 9.8	37.2 ± 7.5	0.101	0.215
Glucose management indicator, %	7.2 ± 0.9	7.0 ± 0.6	7.2 ± 1.0	0.733	0.256
Estimated GFR (MDRD-4, ml/min)	93.8 ± 15.2	99.2 ± 16.2	92.4 ± 14.7	**0**.**028**	0.274
Mean HbA1C DCCT (%)	7.5 ± 1.5	7.5 ± 1.3	7.4 ± 1.5	0.614	0.876
Total cholesterol (mM)	4.6 ± 0.8	4.6 ± 0.7	4.5 ± 0.8	0.847	0.516
Low density lipoprotein cholesterol (mM)	2.6 ± 1.0	2.6 ± 0.6	2.6 ± 0.7	0.807	0.861
High density lipoprotein cholesterol (mM)	1.6 ± 0.4	1.6 ± 0.4	1.6 ± 0.3	0.644	0.541
Triglycerides (mM)	0.7 ± 0.4	0.8 ± 0.5	0.7 ± 0.4	0.082	0.305
Ultrasensitive C-reactive protein (mg/l)	1.6 (0.7, 3.5)	1.9 (0.6, 5.2)	1.6 (0.7, 3.1)	0.440	0.491
Total testosterone (nM)	1.1 ± 0.5	1.6 ± 0.8	1.0 ± 0.4	**<0**.**001**	–
SHBG (nM)	79 (61, 109)	69 (58, 110)	86 (61, 109)	0.203	–
Equilibrium dialysis free testosterone (pM)	7.3 (5.1, 10.7)	10.9 (6.1, 15.9)	6.9 (4.7, 9.9)	**<0**.**001**	–
Calculated free testosterone (pM)	9.8 (7.2, 13.2)	13.2 (8.9, 24.2)	9.2 (6.7, 12.4)	**<0**.**001**	–
Δ^4^-androstenedione (nM)	4.6 ± 2.1	6.7 ± 2.9	4.1 ± 1.4	**<0**.**001**	–
DHEA-S (μM)	4.9 ± 2.2	5.7 ± 2.8	4.7 ± 1.9	0.100	–
17-hydroxyprogesterone (nM)	2.7 ± 1.2	3.0 ± 1.4	2.6 ± 1.1	0.110	–
Prolactin (μg/l)	10.4 (8.2, 14.6)	10.4 (8.1, 15.8)	10.4 (8.2, 13.2)	0.554	–
FSH (UI/l)	5.8 ± 2.6	5.0 ± 1.5	6.1 ± 2.7	**0**.**046**	–
LH (UI/l)	4.7 ± 2.6	6.6 ± 4.1	4.2 ± 1.9	**0**.**003**	–

CSII, continuous subcutaneous insulin infusion; DCCT, diabetes control and complications trial; DHEA-S, dehydroepiandrosterone-sulfate; GDR, glucose disposal rate; GFR, glomerular filtration rate; PCOS, polycystic ovary syndrome; SHBG, sex hormone-binding globulin; T1D, Type 1 diabetes.

Data are shown as number (percentage) for discrete variables and mean ± SD or median and interquartile range (25th percentile, 75th percentile) for continuous variables. Conversion factors to conventional units are provided in the legend of Fig. 2.

Statistically significant differences are shown in bold.

* *P* value was adjusted for age of study and duration of diabetes for metabolic parameters and complications-related variables using regression models.

**Figure 1.**
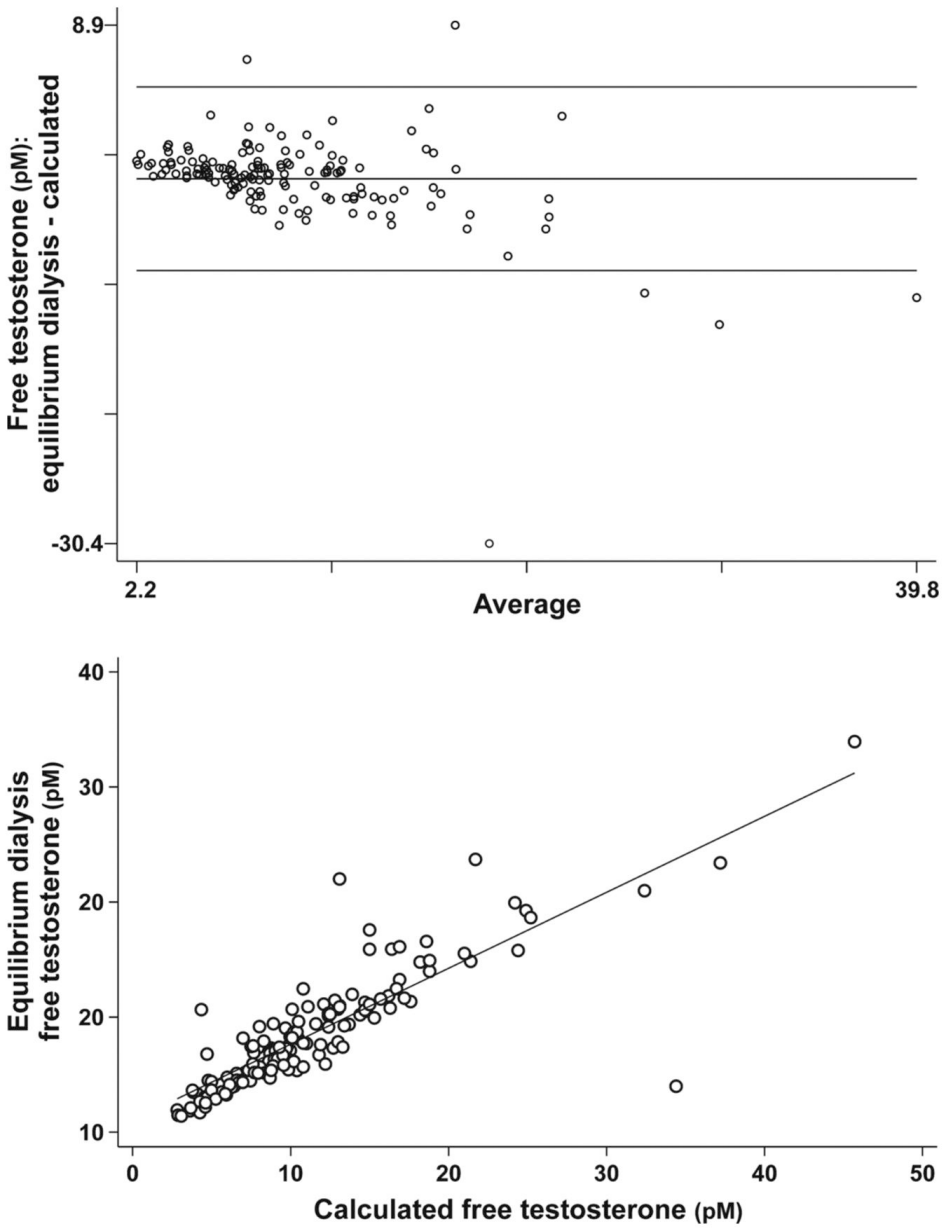
**Concordance between equilibrium dialysis and calculated free testosterone (FT)**. Upper panel figure shows a Bland–Altman plot, with the differences (*y*-axis) plotted against the mean of the two measurements (*x*-axis). The black lines represent the limits of agreement (mean ± 1.96 SD). Bottom panel shows the linear representation of the relationship between the two assays of FT, showing with the line of identity (*y* = *x*). Lin’s Concordance Correlation Coefficient (95% CI) is shown. To convert to metric units, multiply free testosterone by 0.002884 (result in ng/dl).

The agreement between calculated and equilibrium dialysis FT levels was moderate according to a Lin’s CCC of 0.74 (95% CI: 0.68–0.80; Fig. 1). The mean difference between calculated and equilibrium dialysis FT was −2.740 pM, with 95% limits of agreement ranging from −9.561 to 4.082 pM, according to Bland–Altman plots. To perform this analysis, two values of equilibrium dialysis FT that presented extremely high discrepancy with calculated FT (likely related to sample processing issues) were excluded. The 95th percentile of equilibrium dialysis FT in our cohort of T1D women without ovulatory dysfunction nor dermo-cosmetic issues (n = 91) was 18.6 pM.

### PCOS prevalence in T1D

We determined the prevalence of PCOS in women with T1D according to previously described general population reference ranges for androgen measurement when using LC–MS/MS (95th percentile for calculated FT: 23.2 pM) (Luque-Ramírez *et al.*, 2025). PCOS was diagnosed in 30 women, resulting in an overall prevalence of 20% (95% CI: 15–27%). Regarding PCOS phenotypes, 8 women had Phenotype 1 and 7 women had Phenotype 2 (for a total of 15 women with the classic PCOS phenotype), 3 women presented with Phenotype 3 or ovulatory PCOS, and 12 women were diagnosed with Phenotype 4 or non-hyperandrogenic PCOS. Additionally, 28 women presented with menstrual irregularities, 7 with isolated hyperandrogenemia, 6 with isolated PCOM, 1 with idiopathic hirsutism, and 1 with hirsutism and hyperandrogenemia, i.e.: idiopathic hyperandrogenism. In short, functional hyperandrogenism (including idiopathic hyperandrogenism, idiopathic hirsutism, and PCOS) was present in 39 women with T1D (26%, 95% CI: 20–34%). Overall, 43 women from our T1D cohort (29%, 95% CI: 22–37%) presented with any type of reproductive signs or symptoms.

In a second step, we also calculated the prevalence of functional hyperandrogenism and PCOS using, as an upper reference range, the 95th percentile of equilibrium dialysis FT (18.6 pM) derived from our cohort of women with T1D. No woman was reclassified to PCOS or changed PCOS phenotype. Two patients, who were already diagnosed with classic PCOS because of increased TT and oligo-ovulation, had normal calculated FT but increased equilibrium dialysis FT, further confirming both PCOS diagnosis and phenotype. Only one T1D woman who had no reproductive sign or symptom was found to have isolated hyperandrogenemia when using this alternative cut-off value. Accordingly, the prevalence of functional hyperandrogenism increased from 39 to 40 women (27%, 95% CI: 20–35%), while the PCOS prevalence and PCOS phenotype distribution remained unchanged.

### Comparison of T1D women with and without PCOS

We then compared women with T1D and PCOS (n = 30) with those T1D women without PCOS (n = 119). For this comparison, we considered women with T1D and PCOS as a whole, regardless of their particular PCOS phenotype. Characteristics of both groups and the results of the comparisons are also shown in Table 1. Women with T1D and PCOS were younger, had a younger age at the onset of T1D, and presented with a premenarcheal diagnosis of T1D more often than their counterparts without PCOS. Women with T1D and PCOS showed a tendency that was close to reaching statistical significance, to more frequently use continuous subcutaneous insulin infusion (CSII) and to have increased WHRs than those without PCOS, even though BMI was similar in both groups (Table 1). Median hirsutism scores were below 8 in both groups but were higher in women with T1D and PCOS, while there were no significant differences regarding other hyperandrogenic signs such as acne vulgaris or female pattern hair loss (data not shown). There were no differences between the two subgroups of women in AGPs or variables related to diabetes complications.

With respect to laboratory parameters, HbA_1c_ and lipid profiles were similar among the groups. Hormonal analysis showed significantly higher serum TT, calculated and equilibrium dialysis FT, and Δ^4^ concentrations in women with T1D and PCOS when compared to those without PCOS.

### Comparison between women with both T1D and PCOS and typical patients with PCOS

**Figure 2.**
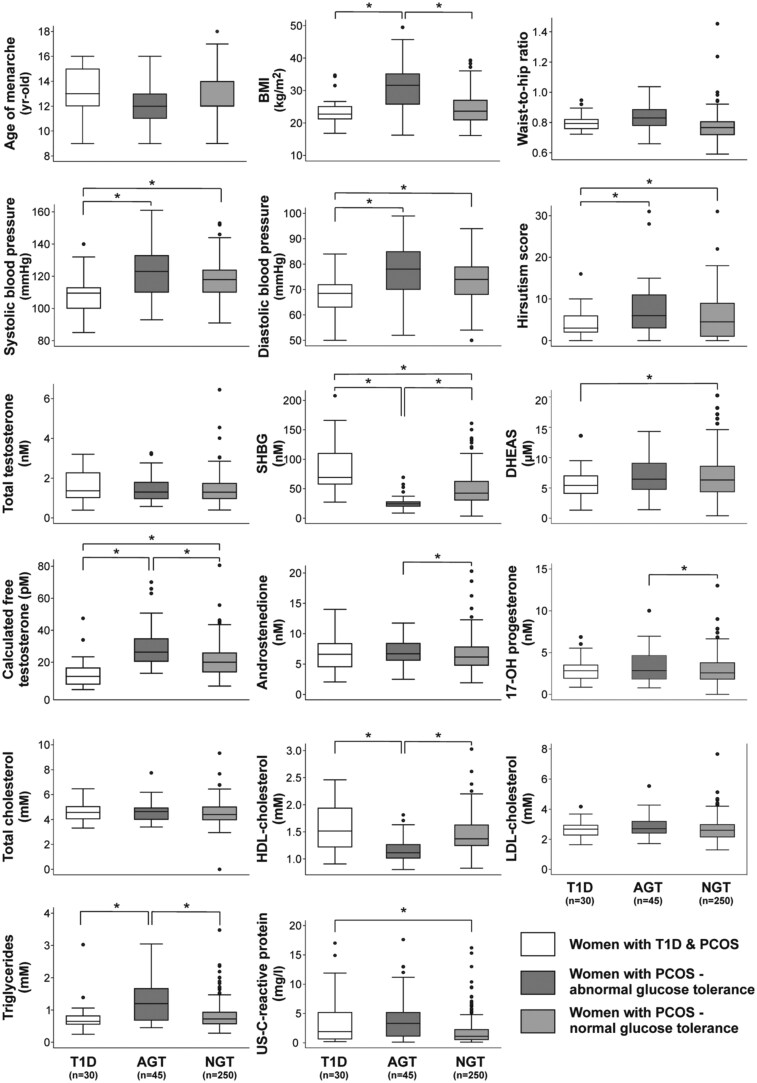
**Box plots representing clinical, hormonal, and other laboratory parameters from the comparison between women with Type 1 diabetes and PCOS (T1D) and typical patients with PCOS presenting with abnormal glucose tolerance (AGT) or normal glucose tolerance (NGT)**. The median is represented by the central line in each box, while the interquartile range is captured by the box edges. Whiskers represent the range of values within 1.5 times the interquartile range, and outliers are displayed as individual points. BMI adjusted *P* values were determined for intergroup comparisons. Statistically significant differences in the pairwise comparisons are represented with an asterisk (*). To convert to conventional units, multiply total testosterone by 28.84 (result in ng/dl), calculated free testosterone by 0.002884 (result in ng/dl), sex hormone-binding globulin (SHBG) by 9 (result in µg/dl), dehydroepiandrosterone-sulfate (DHEAS) by 368.5 (result in ng/ml), Δ^4^-androstendione by 0.2865 (result in ng/ml), 17-OH progesterone by 0.330 (result in ng/ml), cholesterol by 38.6 (result in mg/dl), and triglycerides by 88.5 (result in mg/dl). HDL, high-density lipoproteins; LDL, low-density lipoproteins; US, ultrasensitive.

We then compared the women with T1D and PCOS with age-matched non-T1D patients with PCOS, who presented with either normoglycemia (n = 250), or abnormal glucose tolerance (n = 45) (Table 2 and Fig. 2).

**Table 2.** Comparison between women with T1D and PCOS (n = 30), age-matched women with PCOS and abnormal glucose tolerance (n = 45), and age- and BMI-matched women with PCOS and normoglycemia (n = 250).

Variables	**T1D and PCOS** (n = 30)	**Abnormal glucose tolerance-PCOS** (n = 45)	**Normoglycaemic-PCOS** (n = 250)	*P* value	**Adjusted *P* value** *
Age (years)	25.1 ± 7.1	24.5 ± 4.4	25.4 ± 6.1	0.753	–
Age of menarche (years)^a^	13.3 ± 1.9	12.0 ± 1.9	12.6 ± 1.7	**0.015**	**<0.001**
Family history of PCOS	3 (10.0%)	13 (28.9%)	52 (21.1%)	0.314	0.707
Family history of Type 2 diabetes^b^	21 (70%)	23 (51.1%)	110 (44.7%)	0.070	**0.007**
BMI (kg/m2)^c^	23.6 ± 4.1	31.1 ± 7.3	24.4 ± 4.9	**<0.001**	–
Waist-to-hip ratio^a^	0.80 ± 0.06	0.84 ± 0.09	0.77 ± 0.09	**<0.001**	**<0.001**
Hypertension^b^	1 (3.3%)	20 (47.7%)	79 (32.6%)	**<0.001**	**<0.001**
Systolic blood pressure (mmHg)^b^	110 ± 13	122 ± 15	117 ± 12	**<0.001**	**<0.001**
Diastolic blood pressure (mmHg)^b^	68 ± 8	76 ± 10	74 ± 8	**<0.001**	**<0.001**
Hirsutism score^b^	3 (2, 6)	6 (3, 11)	4.5 (1, 9)	**0.036**	0.333
Hirsutism^b^	2 (6.7%)	20 (44.4%)	82 (33.1%)	**0.001**	0.985
Ovulatory dysfunction	27 (90.0%)	39 (86.7%)	58/76 (76.3%)	0.162	0.746
Total testosterone (nM)	1.58 ± 0.77	1.46 ± 0.66	1.42 ± 0.68	0.463	0.213
Calculated free testosterone (pM)^d^	13.3 (9.0, 24.6)	26.3 (20.4, 34.8)	20.0 (13.8, 26.0)	**<0.001**	**<0.001**
SHBG (nM)^d^	69 (58, 110)	25 (20, 28)	43 (30, 63)	**<0.001**	**<0.001**
Δ^4^-androstenedione (nM)^e^	6.7 ± 2.9	7.1 ± 2.1	6.6 ± 2.6	0.460	**0.002**
DHEA-S (μM)	5.7 ± 2.8	6.8 ± 2.9	6.8 ± 3.4	0.220	0.693
17-hydroxyprogesterone (nM)^e^	3.1 ± 1.5	3.3 ± 2.0	2.9 ± 1.6	0.295	**0.024**
Total cholesterol (mM)	4.6 ± 0.7	4.7 ± 0.9	4.5 ± 0.9	0.409	0.343
Low density lipoprotein-cholesterol (mM)^a^	2.6 ± 0.6	2.9 ± 0.7	2.7 ± 0.7	0.176	**0.003**
High density lipoprotein -cholesterol (mM)^c^	1.6 ± 0.4	1.2 ± 0.2	1.4 ± 0.3	**<0.001**	**<0.001**
Triglycerides (mM)^c^	0.6 (0.6, 0.8)	1.2 (0.7, 1.7)	0.7 (0.6, 0.9)	**<0.001**	**<0.001**
Ultrasensitive C-reactive protein (mg/l)^f^	1.9 (0.6, 5.2)	3.3 (1.1, 5.2)	1.1 (0.5, 2.3)	**<0.001**	**<0.001**

DHEA-S, dehydroepiandrosterone-sulfate; PCOS, polycystic ovary syndrome; SHBG, sex hormone-binding globulin; T1D, Type 1 diabetes.

Data are shown as number (percentage) for discrete variables and mean ± SD or median and interquartile range (25th percentile, 75th percentile) for continuous variables. Conversion factors to conventional units are provided in the legend of Fig. 2.

* *P* value was adjusted for BMI. Statistically significant differences are shown in bold.

a No statistically significant differences were observed in the pairwise comparison between groups.

b Statistically significant differences were observed between T1D & PCOS and abnormal glucose tolerance-PCOS and between T1D and PCOS and normoglycemic-PCOS.

c Statistically significant differences were observed between T1D and PCOS and abnormal glucose tolerance-PCOS and between abnormal glucose tolerance-PCOS and normoglycemic-PCOS.

d Statistically significant differences were observed in the pairwise comparison between the three groups.

e Statistically significant differences were observed between abnormal glucose tolerance-PCOS and normoglycemic-PCOS.

f Statistically significant differences were observed between T1D and PCOS and normoglycemic-PCOS.

Regarding clinical and anthropometric parameters, BMI was significantly higher in patients with PCOS and abnormal glucose tolerance compared to their normoglycemic counterparts and to women with T1D and PCOS. Accordingly, other comparisons among these groups were adjusted by BMI. WHR and age of menarche were similar between the three groups (Fig. 2). Hirsutism was more common in typical patients with PCOS compared with women with both T1D and PCOS, with the largest hirsutism score being observed in those presenting with abnormal glucose tolerance (Fig. 2). TT concentrations were similar in the three groups (Fig. 2). The highest calculated FT levels were observed in typical patients with PCOS and abnormal glucose tolerance, followed by their normoglycemic counterparts and then by women with T1D and PCOS (Fig. 2); these differences were mirrored by SHBG concentrations, which were normal in women with T1D and PCOS but were decreased in typical patients with PCOS, particularly in those with abnormal glucose tolerance (Fig. 2). Circulating DHEA-S concentrations, a marker of adrenal androgen secretion, were increased in normoglycemic women with PCOS compared to those in women with T1D and PCOS (Fig. 2).

Regarding metabolic parameters, high-density lipoprotein cholesterol was reduced and triglycerides were increased in typical patients with PCOS and abnormal glucose tolerance compared to their normoglycemic counterparts and to women with T1D and PCOS (Fig. 2). The prevalence of hypertension was higher in typical patients with PCOS compared to women with T1D and PCOS (Table 2), and systolic BP was higher in patients with PCOS and abnormal glucose tolerance compared to the women with T1D and PCOS (Fig. 2). Finally, ultrasensitive CRP levels were higher in women with T1D and PCOS and patients with PCOS and abnormal glucose tolerance compared to normoglycemic patients with PCOS (Fig. 2).

## Discussion

The results of this study indicate that PCOS is very common in women with T1D reaching a 20% prevalence in our cohort, which is much higher than the ∼6% found in the general population of Spain (Asunción *et al.*, 2000; Sanchón *et al.*, 2012). This is in accordance with other prevalence estimates reported in earlier studies (Bayona *et al.*, 2022) and closest to the 18% prevalence reported in an Irish population using LC–MS/MS for androgen measurements (Gunness *et al.*, 2018). The most common phenotype in our cohort was classic PCOS. Overall, disorders of functional hyperandrogenism, including PCOS, were found in ∼25% of women with T1D in our cohort.

The normal SHBG levels in women with T1D and PCOS may explain their milder hyperandrogenism when compared with women in the general population: hirsutism was not usually present in these women, and their FT was reduced compared to typical women with PCOS despite similar TT levels. We were concerned about a possible misdiagnosis of hyperandrogenemia in earlier studies that relied on indirect calculation of FT in women with T1D (Vermeulen *et al.*, 1999). The concordance between equilibrium dialysis and calculated FT in women with T1D was moderate in our series, with the equilibrium dialysis method yielding lower concentrations compared with calculated FT. Fortunately, when considering equilibrium dialysis FT, the prevalences of PCOS and its phenotypes did not change with regard to those obtained using calculated FT. Hence, calculated FT may be a reasonably accurate method for clinical practice when addressing androgen excess in women with T1D.

Previous studies have shown conflicting results regarding the factors associated with PCOS in women with T1D (Escobar-Morreale and Roldán-Martín, 2016; Bayona *et al.*, 2022; García-Sáenz *et al.*, 2023). A PCOS diagnosis was associated with a younger age at the time of the study, an earlier diabetes onset, and a premenarcheal diagnosis of T1D. Also, more frequent use of CSII and higher daily insulin doses in the subset of T1D patients with PCOS were close to reaching statistical significance. This might support a role for an earlier and longer exposure of the ovaries to supraphysiological amounts of exogenous insulin in the development of PCOS in predisposed women (Escobar-Morreale *et al.*, 2000). Insulin resistance, which might be present in T1D women due to glucotoxicity, abnormal fat mass distribution, and excess weight, may also contribute to worsen exogenous hyperinsulinism by increasing insulin requirements (Fellinger *et al.*, 2019). However, we observed no differences in GDR but, since eGDR is an indirect measure of insulin sensitivity, performing euglycemic hyperinsulinemic clamp in future studies could provide more consistent evidence on this issue.

Overall, these findings are in agreement with the ‘two-hit’ hypothesis we have previously proposed to explain the heterogeneous pathophysiology of PCOS (Escobar-Morreale and San Millán, 2007). The first hit would be an exaggerated capacity for androgen synthesis and secretion, as has been demonstrated at the molecular level using isolated cultures of ovarian theca cells (Wickenheisser *et al.*, 2006) and which may be genetic in origin. Such a defect may be severe enough to cause PCOS during adolescence in some cases; yet, in many patients, the defect becomes apparent only when an acquired second hit aggravates androgen secretion, triggering the development of clinical signs and symptoms (Escobar-Morreale and San Millán, 2007). These triggers include abdominal adiposity, obesity, and insulin resistance, among the best known examples (Escobar-Morreale and San Millán, 2007), although PCOS can also develop in parallel to rarer causes of endogenous hyperinsulinism such as insulinoma or porto-systemic shunt (Murray *et al.*, 2000; Danko *et al.*, 2020). Of note, PCOS resolved in many of these cases when the triggering factor was resolved (Murray *et al.*, 2000; Escobar-Morreale *et al.*, 2005; Danko *et al.*, 2020). Similarly, systemic exogenous hyperinsulinism in women with T1D may trigger PCOS in predisposed women (Roldán *et al.*, 2001; Escobar-Morreale *et al.*, 2021). Also, considering the association of a premenarcheal onset of T1D with the development of PCOS, insulin-like growth factor 1 (IGF-1) could have played some role here. Of note, IGF-1 may also act as a co-gonadotropin at the ovary, and reversible PCOS may develop in women with Laron’s syndrome treated with recombinant IGF-1 (Klinger *et al.*, 1998). However, we found no differences in circulating IGF-1 and its binding proteins 1 and 3, in an early comparison of eight adolescent women with T1D and androgen excess with 29 non-hyperandrogenic counterparts (Roldán, 1999; Roldán *et al.*, 1999).

A few studies have compared women with T1D and PCOS with typical PCOS patients without T1D, with some of them showing similar findings as those found in our cohort (Zachurzok *et al.*, 2013; Amato *et al.*, 2014; Dominic *et al.*, 2023). Other studies, on the contrary, demonstrated no differences in androgen levels between women with T1D and PCOS and BMI-matched non-diabetic women with PCOS (Gunness *et al.*, 2018). The results from these individual studies may be limited by their small sample size and the particular characteristics of the population included in them.

Finally, some differences in metabolic parameters may be noted between women with T1D and PCOS and other PCOS groups. The lower HDL-cholesterol and higher triglyceride concentrations of typical patients with PCOS presenting abnormal glucose tolerance possibly derive from a larger prevalence of metabolic syndrome in these women. Furthermore, the increased ultrasensitive CRP levels in patients with PCOS presenting abnormal glucose tolerance may relate to underlying chronic inflammation (Escobar-Morreale and San Millán, 2007). Also, T1D is a chronic inflammatory disorder that can be worsened by hyperandrogenism, contributing to increased ultrasensitive CRP concentrations.

Among the strengths of our present study, we might highlight the following: (i) the sample size was robust, and patients were recruited consecutively; (ii) we reported the prevalence rates of different phenotypes of PCOS; and (iii) androgen measurements used LC-LC/MS, and by assessing the concordance of equilibrium dialysis and calculated FT in T1D, we ruled out any impact of methodological differences on the estimation of this analyte. As limitations, we acknowledge the possibility of selection bias: having excluded T1D women already diagnosed with PCOS, we may have underestimated actual prevalence rates. In addition, PCOM was described qualitatively in most cases, rather than quantitatively. Moreover, since AMH decreases with age (Piltonen *et al.*, 2005) and we used the same cut off for all women regardless of their age, this aspect could lead to an underdiagnosis of PCOM in women over age 35. Finally, the cross-sectional design of the study precluded us from obtaining any causality insights about the associations found here.

In conclusion, the prevalence of hyperandrogenism and PCOS is increased in premenopausal women with T1D, with the classic combination of hyperandrogenism and ovulatory dysfunction being the most common phenotype. Considering the high frequency of these disorders, their routine screening appears to be warranted. Women with a premenarcheal onset of T1D are particularly susceptible to developing androgen excess disorders and may benefit from future preventive measures at a young age. Hyperandrogenism is relatively mild in women with PCOS and T1D. This may entail a better metabolic profile than those of typical patients with PCOS, but may increase the difficulty in the diagnosis of androgen excess in T1D. Assessing the presence of ovulatory dysfunction may help determine those women at risk, for whom a more complete diagnostic workup, including at least circulating TT and calculated FT concentrations, should be performed.

## Data Availability

De-identified individual patient data and the study protocol are available upon reasonable request to hectorfrancisco.escobar@salud.madrid.org.
